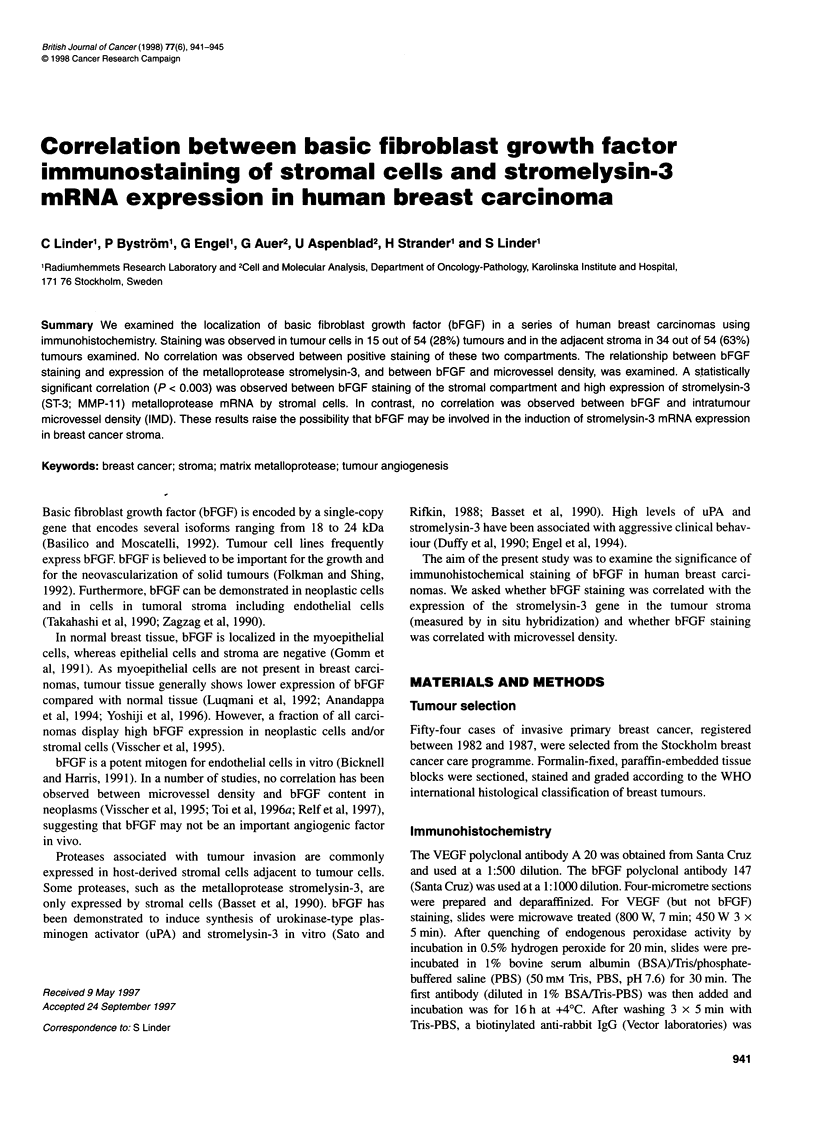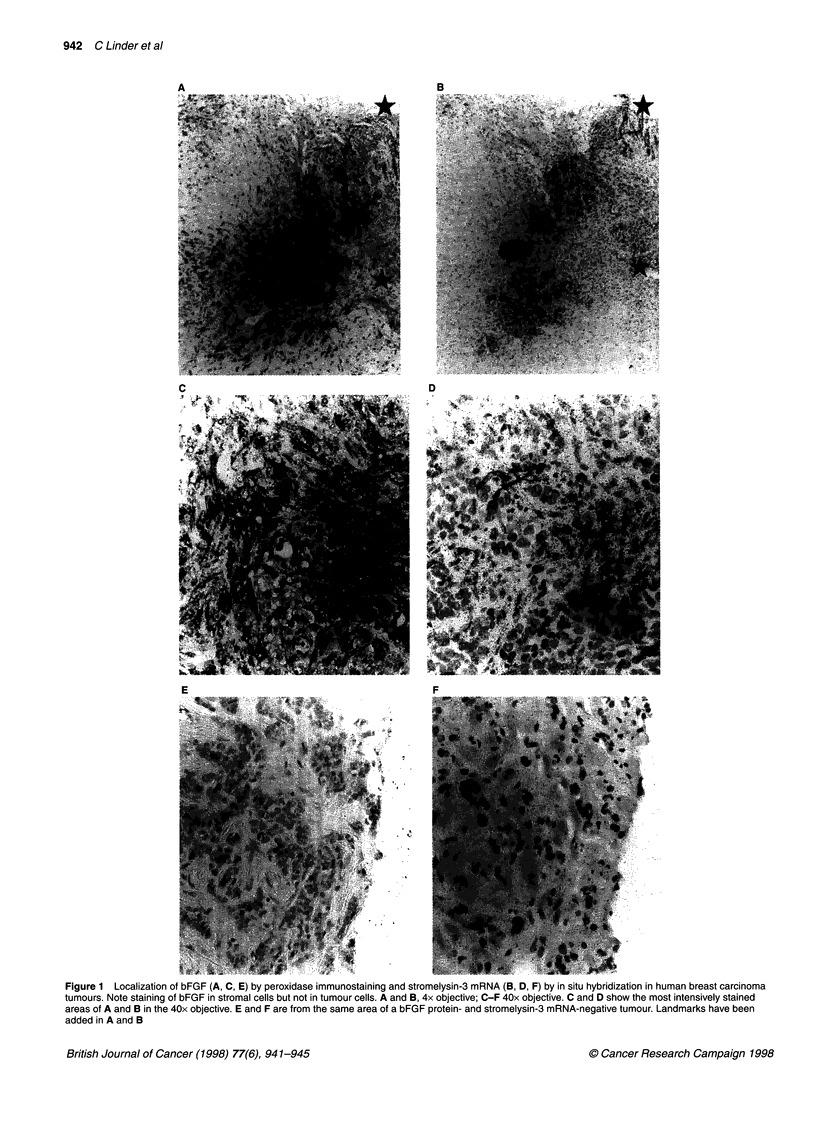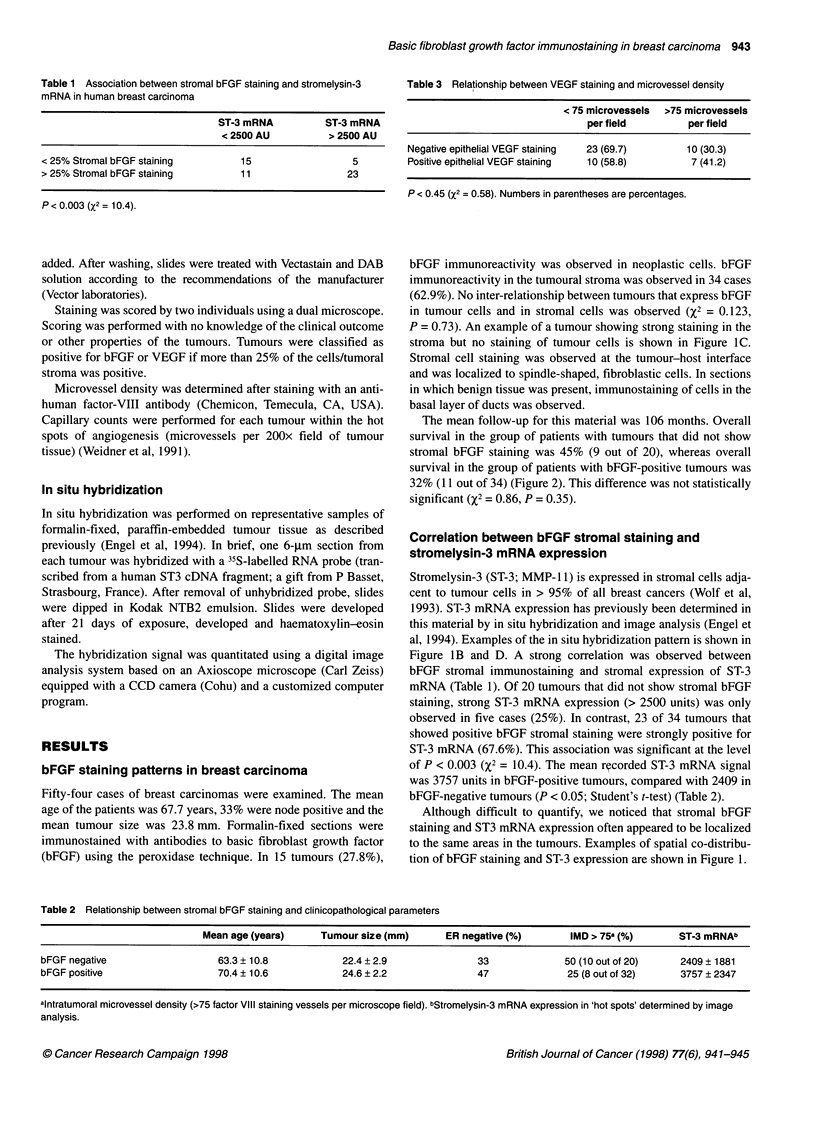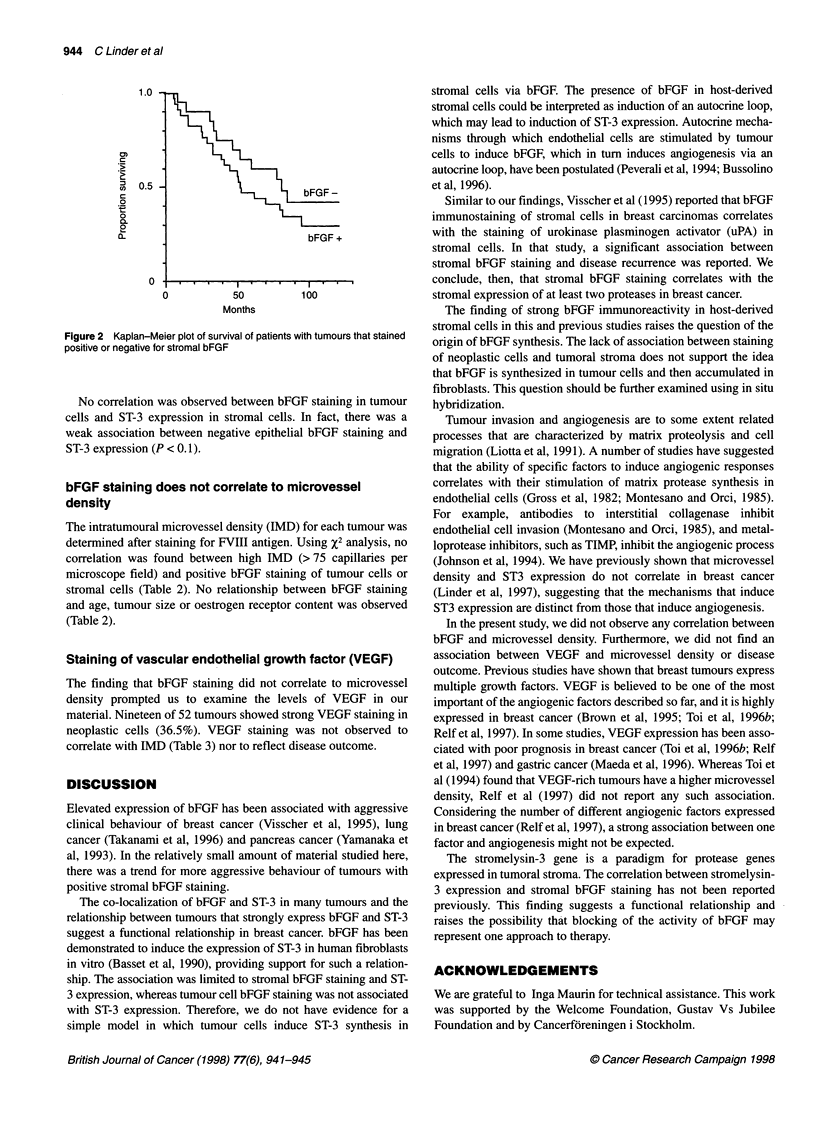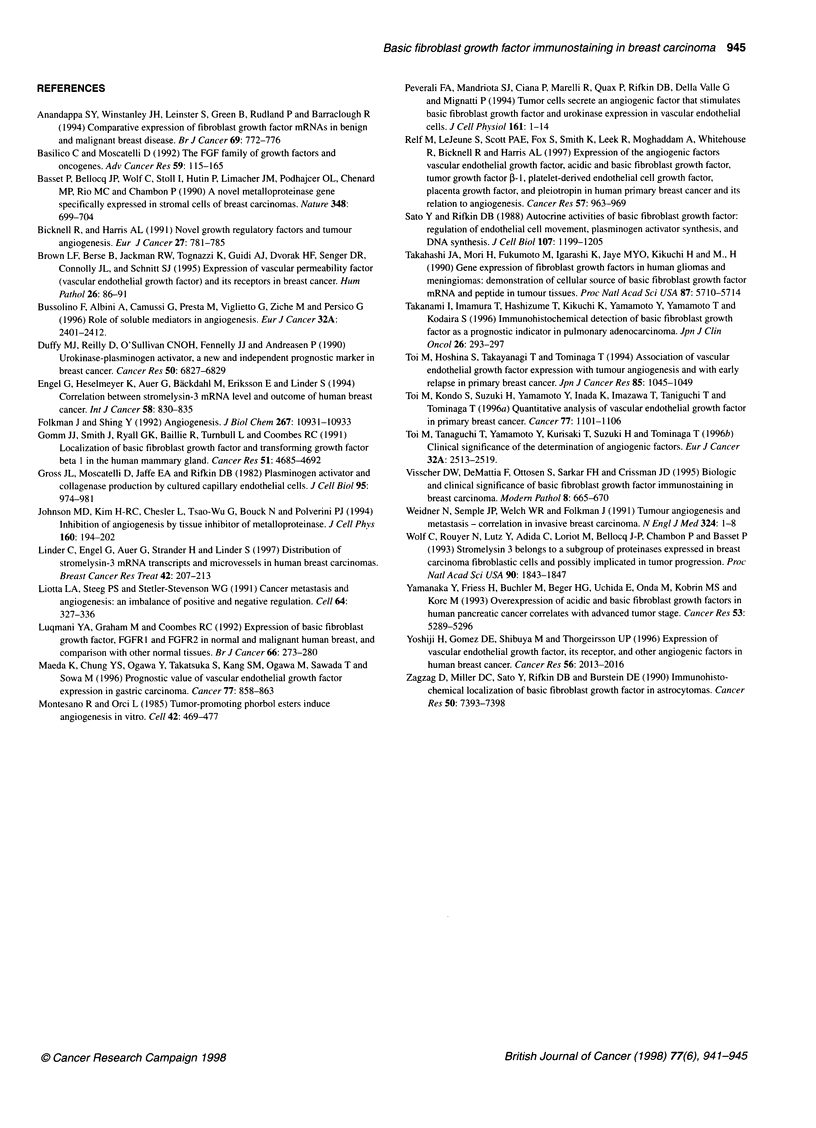# Correlation between basic fibroblast growth factor immunostaining of stromal cells and stromelysin-3 mRNA expression in human breast carcinoma.

**DOI:** 10.1038/bjc.1998.155

**Published:** 1998-03

**Authors:** C. Linder, P. BystrÃ¶m, G. Engel, G. Auer, U. Aspenblad, H. Strander, S. Linder

**Affiliations:** Department of Oncology-Pathology, Karolinska Institute and Hospital, Stockholm, Sweden.

## Abstract

**Images:**


British Journal of Cancer (1998) 77(6), 941–945
© 1998 Cancer Research Campaign

### Correlation between basic fibroblast growth factor immunostaining of stromal cells and stromelysin-3 mRNA expression in human breast carcinoma

**C Linder[1], P Byström[1], G Engel[1], G Auer[2], U Aspenblad[2], H Strander[1] and S Linder[1]**

[1]Radiumhemmets Research Laboratory and [2]Cell and Molecular Analysis, Department of Oncology-Pathology, Karolinska Institute and Hospital, 171 76 Stockholm, Sweden

**Summary** We examined the localization of basic fibroblast growth factor (bFGF) in a series of human breast carcinomas using immunohistochemistry. Staining was observed in tumour cells in 15 out of 54 (28%) tumours and in the adjacent stroma in 34 out of 54 (63%) tumours examined. No correlation was observed between positive staining of these two compartments. The relationship between bFGF staining and expression of the metalloprotease stromelysin-3, and between bFGF and microvessel density, was examined. A statistically significant correlation ($P < 0.003$) was observed between bFGF staining of the stromal compartment and high expression of stromelysin-3 (ST-3; MMP-11) metalloprotease mRNA by stromal cells. In contrast, no correlation was observed between bFGF and intratumour microvessel density (IMD). These results raise the possibility that bFGF may be involved in the induction of stromelysin-3 mRNA expression in breast cancer stroma.

**Keywords:** breast cancer; stroma; matrix metalloprotease; tumour angiogenesis

Basic fibroblast growth factor (bFGF) is encoded by a single-copy gene that encodes several isoforms ranging from 18 to 24 kDa (Basilico and Moscatelli, 1992). Tumour cell lines frequently express bFGF. bFGF is believed to be important for the growth and for the neovascularization of solid tumours (Folkman and Shing, 1992). Furthermore, bFGF can be demonstrated in neoplastic cells and in cells in tumoral stroma including endothelial cells (Takahashi et al, 1990; Zagzag et al, 1990).

In normal breast tissue, bFGF is localized in the myoepithelial cells, whereas epithelial cells and stroma are negative (Gomm et al, 1991). As myoepithelial cells are not present in breast carcinomas, tumour tissue generally shows lower expression of bFGF compared with normal tissue (Luqmani et al, 1992; Anandappa et al, 1994; Yoshiji et al, 1996). However, a fraction of all carcinomas display high bFGF expression in neoplastic cells and/or stromal cells (Visscher et al, 1995).

bFGF is a potent mitogen for endothelial cells in vitro (Bicknell and Harris, 1991). In a number of studies, no correlation has been observed between microvessel density and bFGF content in neoplasms (Visscher et al, 1995; Toi et al, 1996*a*; Relf et al, 1997), suggesting that bFGF may not be an important angiogenic factor in vivo.

Proteases associated with tumour invasion are commonly expressed in host-derived stromal cells adjacent to tumour cells. Some proteases, such as the metalloprotease stromelysin-3, are only expressed by stromal cells (Basset et al, 1990). bFGF has been demonstrated to induce synthesis of urokinase-type plasminogen activator (uPA) and stromelysin-3 in vitro (Sato and Rifkin, 1988; Basset et al, 1990). High levels of uPA and stromelysin-3 have been associated with aggressive clinical behaviour (Duffy et al, 1990; Engel et al, 1994).

The aim of the present study was to examine the significance of immunohistochemical staining of bFGF in human breast carcinomas. We asked whether bFGF staining was correlated with the expression of the stromelysin-3 gene in the tumour stroma (measured by in situ hybridization) and whether bFGF staining was correlated with microvessel density.

*Received 9 May 1997*
*Accepted 24 September 1997*

*Correspondence to:* S Linder

### MATERIALS AND METHODS

### Tumour selection

Fifty-four cases of invasive primary breast cancer, registered between 1982 and 1987, were selected from the Stockholm breast cancer care programme. Formalin-fixed, paraffin-embedded tissue blocks were sectioned, stained and graded according to the WHO international histological classification of breast tumours.

### Immunohistochemistry

The VEGF polyclonal antibody A 20 was obtained from Santa Cruz and used at a 1:500 dilution. The bFGF polyclonal antibody 147 (Santa Cruz) was used at a 1:1000 dilution. Four-micrometre sections were prepared and deparaffinized. For VEGF (but not bFGF) staining, slides were microwave treated (800 W, 7 min; 450 W 3 × 5 min). After quenching of endogenous peroxidase activity by incubation in 0.5% hydrogen peroxide for 20 min, slides were pre-incubated in 1% bovine serum albumin (BSA)/Tris/phosphate-buffered saline (PBS) (50 mM Tris, PBS, pH 7.6) for 30 min. The first antibody (diluted in 1% BSA/Tris-PBS) was then added and incubation was for 16 h at +4°C. After washing 3 × 5 min with Tris-PBS, a biotinylated anti-rabbit IgG (Vector laboratories) was

**Figure 1** Localization of bFGF (**A**, **C**, **E**) by peroxidase immunostaining and stromelysin-3 mRNA (**B**, **D**, **F**) by in situ hybridization in human breast carcinoma tumours. Note staining of bFGF in stromal cells but not in tumour cells. **A** and **B**, 4× objective; **C–F** 40× objective. **C** and **D** show the most intensively stained areas of **A** and **B** in the 40× objective. **E** and **F** are from the same area of a bFGF protein- and stromelysin-3 mRNA-negative tumour. Landmarks have been added in **A** and **B**

 © Cancer Research Campaign 1998

**Table 1** Association between stromal bFGF staining and stromelysin-3 mRNA in human breast carcinoma

| | ST-3 mRNA < 2500 AU | ST-3 mRNA > 2500 AU |
|---|---|---|
| < 25% Stromal bFGF staining | 15 | 5 |
| > 25% Stromal bFGF staining | 11 | 23 |

$P < 0.003$ ($\chi^2 = 10.4$).

**Table 3** Relationship between VEGF staining and microvessel density

| | < 75 microvessels per field | >75 microvessels per field |
|---|---|---|
| Negative epithelial VEGF staining | 23 (69.7) | 10 (30.3) |
| Positive epithelial VEGF staining | 10 (58.8) | 7 (41.2) |

$P < 0.45$ ($\chi^2 = 0.58$). Numbers in parentheses are percentages.

added. After washing, slides were treated with Vectastain and DAB solution according to the recommendations of the manufacturer (Vector laboratories).

Staining was scored by two individuals using a dual microscope. Scoring was performed with no knowledge of the clinical outcome or other properties of the tumours. Tumours were classified as positive for bFGF or VEGF if more than 25% of the cells/tumoral stroma was positive.

Microvessel density was determined after staining with an anti-human factor-VIII antibody (Chemicon, Temecula, CA, USA). Capillary counts were performed for each tumour within the hot spots of angiogenesis (microvessels per 200× field of tumour tissue) (Weidner et al, 1991).

### In situ hybridization

In situ hybridization was performed on representative samples of formalin-fixed, paraffin-embedded tumour tissue as described previously (Engel et al, 1994). In brief, one 6-µm section from each tumour was hybridized with a $^{35}S$-labelled RNA probe (transcribed from a human ST3 cDNA fragment; a gift from P Basset, Strasbourg, France). After removal of unhybridized probe, slides were dipped in Kodak NTB2 emulsion. Slides were developed after 21 days of exposure, developed and haematoxylin–eosin stained.

The hybridization signal was quantitated using a digital image analysis system based on an Axioscope microscope (Carl Zeiss) equipped with a CCD camera (Cohu) and a customized computer program.

### RESULTS

### bFGF staining patterns in breast carcinoma

Fifty-four cases of breast carcinomas were examined. The mean age of the patients was 67.7 years, 33% were node positive and the mean tumour size was 23.8 mm. Formalin-fixed sections were immunostained with antibodies to basic fibroblast growth factor (bFGF) using the peroxidase technique. In 15 tumours (27.8%), bFGF immunoreactivity was observed in neoplastic cells. bFGF immunoreactivity in the tumoural stroma was observed in 34 cases (62.9%). No inter-relationship between tumours that express bFGF in tumour cells and in stromal cells was observed ($\chi^2 = 0.123$, $P = 0.73$). An example of a tumour showing strong staining in the stroma but no staining of tumour cells is shown in Figure 1C. Stromal cell staining was observed at the tumour–host interface and was localized to spindle-shaped, fibroblastic cells. In sections in which benign tissue was present, immunostaining of cells in the basal layer of ducts was observed.

The mean follow-up for this material was 106 months. Overall survival in the group of patients with tumours that did not show stromal bFGF staining was 45% (9 out of 20), whereas overall survival in the group of patients with bFGF-positive tumours was 32% (11 out of 34) (Figure 2). This difference was not statistically significant ($\chi^2 = 0.86$, $P = 0.35$).

### Correlation between bFGF stromal staining and stromelysin-3 mRNA expression

Stromelysin-3 (ST-3; MMP-11) is expressed in stromal cells adjacent to tumour cells in > 95% of all breast cancers (Wolf et al, 1993). ST-3 mRNA expression has previously been determined in this material by in situ hybridization and image analysis (Engel et al, 1994). Examples of the in situ hybridization pattern is shown in Figure 1B and D. A strong correlation was observed between bFGF stromal immunostaining and stromal expression of ST-3 mRNA (Table 1). Of 20 tumours that did not show stromal bFGF staining, strong ST-3 mRNA expression (> 2500 units) was only observed in five cases (25%). In contrast, 23 of 34 tumours that showed positive bFGF stromal staining were strongly positive for ST-3 mRNA (67.6%). This association was significant at the level of $P < 0.003$ ($\chi^2 = 10.4$). The mean recorded ST-3 mRNA signal was 3757 units in bFGF-positive tumours, compared with 2409 in bFGF-negative tumours ($P < 0.05$; Student's $t$-test) (Table 2).

Although difficult to quantify, we noticed that stromal bFGF staining and ST3 mRNA expression often appeared to be localized to the same areas in the tumours. Examples of spatial co-distribution of bFGF staining and ST-3 expression are shown in Figure 1.

**Table 2** Relationship between stromal bFGF staining and clinicopathological parameters

| | Mean age (years) | Tumour size (mm) | ER negative (%) | IMD > 75[a] (%) | ST-3 mRNA[b] |
|---|---|---|---|---|---|
| bFGF negative | 63.3 ± 10.8 | 22.4 ± 2.9 | 33 | 50 (10 out of 20) | 2409 ± 1881 |
| bFGF positive | 70.4 ± 10.6 | 24.6 ± 2.2 | 47 | 25 (8 out of 32) | 3757 ± 2347 |

[a]Intratumoral microvessel density (>75 factor VIII staining vessels per microscope field). [b]Stromelysin-3 mRNA expression in 'hot spots' determined by image analysis.

© Cancer Research Campaign 1998

**Figure 2** Kaplan–Meier plot of survival of patients with tumours that stained positive or negative for stromal bFGF

No correlation was observed between bFGF staining in tumour cells and ST-3 expression in stromal cells. In fact, there was a weak association between negative epithelial bFGF staining and ST-3 expression ($P < 0.1$).

### bFGF staining does not correlate to microvessel density

The intratumoural microvessel density (IMD) for each tumour was determined after staining for FVIII antigen. Using $\chi^2$ analysis, no correlation was found between high IMD (> 75 capillaries per microscope field) and positive bFGF staining of tumour cells or stromal cells (Table 2). No relationship between bFGF staining and age, tumour size or oestrogen receptor content was observed (Table 2).

### Staining of vascular endothelial growth factor (VEGF)

The finding that bFGF staining did not correlate to microvessel density prompted us to examine the levels of VEGF in our material. Nineteen of 52 tumours showed strong VEGF staining in neoplastic cells (36.5%). VEGF staining was not observed to correlate with IMD (Table 3) nor to reflect disease outcome.

### DISCUSSION

Elevated expression of bFGF has been associated with aggressive clinical behaviour of breast cancer (Visscher et al, 1995), lung cancer (Takanami et al, 1996) and pancreas cancer (Yamanaka et al, 1993). In the relatively small amount of material studied here, there was a trend for more aggressive behaviour of tumours with positive stromal bFGF staining.

The co-localization of bFGF and ST-3 in many tumours and the relationship between tumours that strongly express bFGF and ST-3 suggest a functional relationship in breast cancer. bFGF has been demonstrated to induce the expression of ST-3 in human fibroblasts in vitro (Basset et al, 1990), providing support for such a relationship. The association was limited to stromal bFGF staining and ST-3 expression, whereas tumour cell bFGF staining was not associated with ST-3 expression. Therefore, we do not have evidence for a simple model in which tumour cells induce ST-3 synthesis in stromal cells via bFGF. The presence of bFGF in host-derived stromal cells could be interpreted as induction of an autocrine loop, which may lead to induction of ST-3 expression. Autocrine mechanisms through which endothelial cells are stimulated by tumour cells to induce bFGF, which in turn induces angiogenesis via an autocrine loop, have been postulated (Peverali et al, 1994; Bussolino et al, 1996).

Similar to our findings, Visscher et al (1995) reported that bFGF immunostaining of stromal cells in breast carcinomas correlates with the staining of urokinase plasminogen activator (uPA) in stromal cells. In that study, a significant association between stromal bFGF staining and disease recurrence was reported. We conclude, then, that stromal bFGF staining correlates with the stromal expression of at least two proteases in breast cancer.

The finding of strong bFGF immunoreactivity in host-derived stromal cells in this and previous studies raises the question of the origin of bFGF synthesis. The lack of association between staining of neoplastic cells and tumoral stroma does not support the idea that bFGF is synthesized in tumour cells and then accumulated in fibroblasts. This question should be further examined using in situ hybridization.

Tumour invasion and angiogenesis are to some extent related processes that are characterized by matrix proteolysis and cell migration (Liotta et al, 1991). A number of studies have suggested that the ability of specific factors to induce angiogenic responses correlates with their stimulation of matrix protease synthesis in endothelial cells (Gross et al, 1982; Montesano and Orci, 1985). For example, antibodies to interstitial collagenase inhibit endothelial cell invasion (Montesano and Orci, 1985), and metalloprotease inhibitors, such as TIMP, inhibit the angiogenic process (Johnson et al, 1994). We have previously shown that microvessel density and ST3 expression do not correlate in breast cancer (Linder et al, 1997), suggesting that the mechanisms that induce ST3 expression are distinct from those that induce angiogenesis.

In the present study, we did not observe any correlation between bFGF and microvessel density. Furthermore, we did not find an association between VEGF and microvessel density or disease outcome. Previous studies have shown that breast tumours express multiple growth factors. VEGF is believed to be one of the most important of the angiogenic factors described so far, and it is highly expressed in breast cancer (Brown et al, 1995; Toi et al, 1996*b*; Relf et al, 1997). In some studies, VEGF expression has been associated with poor prognosis in breast cancer (Toi et al, 1996*b*; Relf et al, 1997) and gastric cancer (Maeda et al, 1996). Whereas Toi et al (1994) found that VEGF-rich tumours have a higher microvessel density, Relf et al (1997) did not report any such association. Considering the number of different angiogenic factors expressed in breast cancer (Relf et al, 1997), a strong association between one factor and angiogenesis might not be expected.

The stromelysin-3 gene is a paradigm for protease genes expressed in tumoral stroma. The correlation between stromelysin-3 expression and stromal bFGF staining has not been reported previously. This finding suggests a functional relationship and raises the possibility that blocking of the activity of bFGF may represent one approach to therapy.

### ACKNOWLEDGEMENTS

We are grateful to Inga Maurin for technical assistance. This work was supported by the Welcome Foundation, Gustav Vs Jubilee Foundation and by Cancerföreningen i Stockholm.

© Cancer Research Campaign 1998

### REFERENCES

Anandappa SY, Winstanley JH, Leinster S, Green B, Rudland P and Barraclough R (1994) Comparative expression of fibroblast growth factor mRNAs in benign and malignant breast disease. *Br J Cancer* **69**: 772–776

Basilico C and Moscatelli D (1992) The FGF family of growth factors and oncogenes. *Adv Cancer Res* **59**: 115–165

Basset P, Bellocq JP, Wolf C, Stoll I, Hutin P, Limacher JM, Podhajcer OL, Chenard MP, Rio MC and Chambon P (1990) A novel metalloproteinase gene specifically expressed in stromal cells of breast carcinomas. *Nature* **348**: 699–704

Bicknell R, and Harris AL (1991) Novel growth regulatory factors and tumour angiogenesis. *Eur J Cancer* **27**: 781–785

Brown LF, Berse B, Jackman RW, Tognazzi K, Guidi AJ, Dvorak HF, Senger DR, Connolly JL, and Schnitt SJ (1995) Expression of vascular permeability factor (vascular endothelial growth factor) and its receptors in breast cancer. *Hum Pathol* **26**: 86–91

Bussolino F, Albini A, Camussi G, Presta M, Viglietto G, Ziche M and Persico G (1996) Role of soluble mediators in angiogenesis. *Eur J Cancer* **32A**: 2401–2412.

Duffy MJ, Reilly D, O'Sullivan CNOH, Fennelly JJ and Andreasen P (1990) Urokinase-plasminogen activator, a new and independent prognostic marker in breast cancer. *Cancer Res* **50**: 6827–6829

Engel G, Heselmeyer K, Auer G, Bäckdahl M, Eriksson E and Linder S (1994) Correlation between stromelysin-3 mRNA level and outcome of human breast cancer. *Int J Cancer* **58**: 830–835

Folkman J and Shing Y (1992) Angiogenesis. *J Biol Chem* **267**: 10931–10933

Gomm JJ, Smith J, Ryall GK, Baillie R, Turnbull L and Coombes RC (1991) Localization of basic fibroblast growth factor and transforming growth factor beta 1 in the human mammary gland. *Cancer Res* **51**: 4685–4692

Gross JL, Moscatelli D, Jaffe EA and Rifkin DB (1982) Plasminogen activator and collagenase production by cultured capillary endothelial cells. *J Cell Biol* **95**: 974–981

Johnson MD, Kim H-RC, Chesler L, Tsao-Wu G, Bouck N and Polverini PJ (1994) Inhibition of angiogenesis by tissue inhibitor of metalloproteinase. *J Cell Phys* **160**: 194–202

Linder C, Engel G, Auer G, Strander H and Linder S (1997) Distribution of stromelysin-3 mRNA transcripts and microvessels in human breast carcinomas. *Breast Cancer Res Treat* **42**: 207–213

Liotta LA, Steeg PS and Stetler-Stevenson WG (1991) Cancer metastasis and angiogenesis: an imbalance of positive and negative regulation. *Cell* **64**: 327–336

Luqmani YA, Graham M and Coombes RC (1992) Expression of basic fibroblast growth factor, FGFR1 and FGFR2 in normal and malignant human breast, and comparison with other normal tissues. *Br J Cancer* **66**: 273–280

Maeda K, Chung YS, Ogawa Y, Takatsuka S, Kang SM, Ogawa M, Sawada T and Sowa M (1996) Prognostic value of vascular endothelial growth factor expression in gastric carcinoma. *Cancer* **77**: 858–863

Montesano R and Orci L (1985) Tumor-promoting phorbol esters induce angiogenesis in vitro. *Cell* **42**: 469–477

Peverali FA, Mandriota SJ, Ciana P, Marelli R, Quax P, Rifkin DB, Della Valle G and Mignatti P (1994) Tumor cells secrete an angiogenic factor that stimulates basic fibroblast growth factor and urokinase expression in vascular endothelial cells. *J Cell Physiol* **161**: 1–14

Relf M, LeJeune S, Scott PAE, Fox S, Smith K, Leek R, Moghaddam A, Whitehouse R, Bicknell R and Harris AL (1997) Expression of the angiogenic factors vascular endothelial growth factor, acidic and basic fibroblast growth factor, tumor growth factor β-1, platelet-derived endothelial cell growth factor, placenta growth factor, and pleiotropin in human primary breast cancer and its relation to angiogenesis. *Cancer Res* **57**: 963–969

Sato Y and Rifkin DB (1988) Autocrine activities of basic fibroblast growth factor: regulation of endothelial cell movement, plasminogen activator synthesis, and DNA synthesis. *J Cell Biol* **107**: 1199–1205

Takahashi JA, Mori H, Fukumoto M, Igarashi K, Jaye MYO, Kikuchi H and M., H (1990) Gene expression of fibroblast growth factors in human gliomas and meningiomas: demonstration of cellular source of basic fibroblast growth factor mRNA and peptide in tumour tissues. *Proc Natl Acad Sci USA* **87**: 5710–5714

Takanami I, Imamura T, Hashizume T, Kikuchi K, Yamamoto Y, Yamamoto T and Kodaira S (1996) Immunohistochemical detection of basic fibroblast growth factor as a prognostic indicator in pulmonary adenocarcinoma. *Jpn J Clin Oncol* **26**: 293–297

Toi M, Hoshina S, Takayanagi T and Tominaga T (1994) Association of vascular endothelial growth factor expression with tumour angiogenesis and with early relapse in primary breast cancer. *Jpn J Cancer Res* **85**: 1045–1049

Toi M, Kondo S, Suzuki H, Yamamoto Y, Inada K, Imazawa T, Taniguchi T and Tominaga T (1996*a*) Quantitative analysis of vascular endothelial growth factor in primary breast cancer. *Cancer* **77**: 1101–1106

Toi M, Tanaguchi T, Yamamoto Y, Kurisaki T, Suzuki H and Tominaga T (1996*b*) Clinical significance of the determination of angiogenic factors. *Eur J Cancer* **32A**: 2513–2519.

Visscher DW, DeMattia F, Ottosen S, Sarkar FH and Crissman JD (1995) Biologic and clinical significance of basic fibroblast growth factor immunostaining in breast carcinoma. *Modern Pathol* **8**: 665–670

Weidner N, Semple JP, Welch WR and Folkman J (1991) Tumour angiogenesis and metastasis – correlation in invasive breast carcinoma. *N Engl J Med* **324**: 1–8

Wolf C, Rouyer N, Lutz Y, Adida C, Loriot M, Bellocq J-P, Chambon P and Basset P (1993) Stromelysin 3 belongs to a subgroup of proteinases expressed in breast carcinoma fibroblastic cells and possibly implicated in tumor progression. *Proc Natl Acad Sci USA* **90**: 1843–1847

Yamanaka Y, Friess H, Buchler M, Beger HG, Uchida E, Onda M, Kobrin MS and Korc M (1993) Overexpression of acidic and basic fibroblast growth factors in human pancreatic cancer correlates with advanced tumor stage. *Cancer Res* **53**: 5289–5296

Yoshiji H, Gomez DE, Shibuya M and Thorgeirsson UP (1996) Expression of vascular endothelial growth factor, its receptor, and other angiogenic factors in human breast cancer. *Cancer Res* **56**: 2013–2016

Zagzag D, Miller DC, Sato Y, Rifkin DB and Burstein DE (1990) Immunohistochemical localization of basic fibroblast growth factor in astrocytomas. *Cancer Res* **50**: 7393–7398

© Cancer Research Campaign 1998